# *Clostridium perfringens* Type A Isolated from Intestinal Contents of Alpaca

**DOI:** 10.3390/microorganisms14010166

**Published:** 2026-01-12

**Authors:** Hongrui Ren, Qiong Jia, Shuaipeng Gao, Haoyu Yang, Shuyin Zhang, Ruiwen Fan

**Affiliations:** 1 College of Veterinary Medicine, Shanxi Agricultural University, Jingzhong 030801, China; ren18740121@163.com (H.R.); jiaqiong7270@163.com (Q.J.); mikulove23333@gmail.com (S.G.); y1208436381y@163.com (H.Y.); z15713597719@163.com (S.Z.); 2 Xinjiang Laboratory of Special Environmental Microbiology, Institute of Microbiology, Xinjiang Academy of Agricultural Sciences, Urumqi 830091, China; 3 College of Agriculture and Biology, Liaocheng University, Liaocheng 252000, China

**Keywords:** α toxin, *Clostridium perfringens*, Occludin, T lymphocytes, ZO-1

## Abstract

*C. perfringens* is a Gram-positive, anaerobic, spore-forming bacterium that causes serious diseases in humans and animals. In this study, *C. perfringens* was isolated from the intestinal content of an alpaca, cultured, and then identified using laboratory methods including Gram staining, biochemical tests, and PCR for *16S rRNA* of six toxins. Furthermore, the pathogenicity of different strains was analyzed in mice. The results showed that *C. perfringens* was identified as type A and caused severe pathology of the spleen, lungs, and duodenum in mice through CD4^+^ and CD8^+^ T cells. Also, the mRNA expression levels of ZO-1 and Occludin were further quantified by qRT-PCR with normalization to β-actin, which showed decreased expression levels in the duodenum of mice in the gavage group compared to those in the NC groups, with significant differences (*n* = 3; * *p* < 0.05, ** *p* < 0.01). The results could inform the development of drugs and vaccines resistant to *C. perfringens* in alpaca.

## Simple Summary

A *Clostridium perfringens* (*C. perfringens*) type A strain was isolated and identified from a dead alpaca’s intestine. The pathogenicity of *C. perfringens* in mice was then determined using the intragastric method with a bacterial supernatant and showed severe duodenal pathology, including epithelial basement membrane detachment and splenic white pulp dilation with the dominant distribution of CD8^+^ T cells. Furthermore, the intestinal barrier was disrupted by the decreased expressions of ZO-1 and Occludin in the duodenal mucosa. The results indicate that the α toxin produced by the type A strain triggers CD8^+^ T cell activation, aggravating immune response and mucosal damage.

## 1. Introduction

*Clostridium perfringens* (*C. perfringens*) is a Gram-positive, anaerobic, spore-forming bacterium that causes a variety of diseases in humans and other animals. *C. perfringens* can produce four major toxins (α, β, ε, and ι) and is divided into five serotypes, from A to E [1]. The α toxin produced by the type A strain is the major virulence factor of *C. perfringens* [2] and has been identified as the principal toxin involved in gas gangrene and bovine necrohemorrhagic enteritis [2]. The type B strain, which produces α, β, and ε toxins, is the etiologic agent of dysentery in newborn lambs and hemorrhagic enteritis and enterotoxemia in goats, calves, and foals [3]. The type C strain causes highly lethal diseases in many mammalian species that are characterized mainly by necrotizing enteritis or enterocolitis [4]. Neonates are particularly susceptible to β toxin. The type D strain mainly causes infections in goats and calves. The type E strain produces both α and ι toxins, but the mechanism of its effect has not been fully elucidated.

Alpaca (*Vicugna pacos*) is an economically important South American camelid (SAC) species bred around the world for its meat, for its fur, and as a companion and nanobody-producing animal [5]. However, the number of alpacas has decreased slowly because of inherited reproductive characteristics and disease. In China, infections caused by *C. perfringens* in baby alpaca are characterized by occult occurrence, a short natural course, and high mortality, which results in considerable financial losses for farmers and the livestock industry [6]. In this study, *C. perfringens* was isolated from the intestinal contents of a dead alpaca, cultured, and then identified using laboratory methods include Gram staining, biochemical tests, and PCR. Furthermore, the pathogenicity of the toxin produced by the strain was tested in mice. The results will be beneficial for the treatment and the development of a vaccine for *C. perfringens* in alpaca and other ruminant animals.

## 2. Materials and Methods

### 2.1. Isolation and Purification of Strains

The visceral organs of a 6-month-old dead alpaca were inoculated and streaked onto a Tryptose–Sulfite–Cycloserine (TSC) plate, and incubated in an anaerobic tank with anaerobic package at 37 °C for 24 h. Single colonies were then selected from the plate, inoculated into fluid thioglycollate medium (FTG), and incubated at 37 °C for 12 h. The single colonies were then picked for purification. The purified strain was deposited and named as “*Clostridium perfringens* strain SXAU-alpaca.”

### 2.2. Gram Identification and Biochemical Testing

The isolated bacteria were smeared onto slides, fixed by heat, stained with Gram staining solution for 3.5 min, and observed under oil immersion using a light microscope (×1000). Biochemical testing of the isolated strains was carried out using bacterial biochemical tubes with lactose–gelatin, iron milk medium, and nitrate reduction (Qingdao haibo Biological, Qingdao, China).

### 2.3. PCR Identification

Genomic DNA of the purified strains was isolated using the Gram-positive Bacterial Genomic DNA kit (Solarbio, Beijing, China) following the instructions. The bacterial *16S rRNA* gene was amplified via the PCR method using the isolated DNA as a template. The PCR product was identified by sequencing (Sangon, Shanghai, China). The strains were genotyped for types A, B, C, D, and E using multiplex PCR. A total of 50 μL of PCR reaction mixture, including template DNA (200 ng), 0.4 μM of each primer (α, β, ε, ι, CPE, Net B), 2 × PCR Mix 25 μL, and ddH_2_O, was run in the thermocycle, starting with initial denaturation at 95 °C for 5 min; followed by 35 cycles at 95 °C for 30 s and 58 °C for 30 s, and then extension at 72 °C for 1 min (the primers are listed in Table 1). The PCR products were separated via 1% agarose gel electrophoresis and sequenced, and then, the phylogenetic tree was constructed using the software MEGA 11.0 and the neighbor-joining method.

### 2.4. Animal Pathogenicity

The purified strain was inoculated into FTG medium and incubated overnight at 37 °C in an anaerobic tank containing an anaerobic package with a bacterial concentration of 5 × 10^8^ cfu/mL. The bacterial liquid was centrifuged at 8000× *g* for 15 min to obtain the supernatant, which was then filtered through a 0.22 μm filter. Healthy 20 6-week-old Balb/c mice with similar body weights were selected and randomly divided into 2 groups with 10 mice in each group (5 males and 5 females) for gavage. According to the trial experiment, 300 μL of the prepared bacterial supernatant and filtered FTG blank culture medium was then administered to each mouse by gavage. After 7 d of continuous gavage, the mice were sacrificed, and their spleen, lungs, and duodenum were taken and fixed in Bouin’s solution for hematoxylin and eosin (H & E) staining, immunofluorescence, and immunohistochemistry.

### 2.5. Immunofluorescence (IFC)

Paraffin sections were dewaxed and hydrated using gradient alcohol. Additionally, the antigen was retrieved with sodium citrate for 20 min. Sections were then blocked with 3% bovine serum albumin (BSA) at 37 °C for 30 min and incubated at 4 °C overnight using a rabbit anti-CD4 antibody (1:400, Proteintech, Wuhan, China) and mouse anti-CD8 antibody (1:400, Bioss, Beijing, China) mixture solution. After three washes with PBS at 5 min each, the sections were then incubated with FITC-conjugated goat anti-mouse IgG and PE-conjugated goat anti-rabbit IgG for 30 min. Following nuclear DAPI staining for 10 min, the sections were sealed with an antifluorescence quenching agent, and the slides were observed under a fluorescence microscope.

### 2.6. Immunohistochemistry (IHC)

After dewaxing and antigen repair, the paraffin sections of the duodenum were incubated at 3% H_2_O_2_ room temperature for 20 min to remove endogenous peroxidase. Then, the sections were washed with PBS three times and blocked in 3% BSA 37 °C for 30 min. The sections were incubated in mouse anti-ZO-1 antibody (Proteintech, Wuhan, China) or anti-Occludin antibody (Proteintech, Wuhan, China) diluted in phosphate-buffered solution (1:400) at 4 °C overnight. After washing in PBS, the sections were incubated with goat anti-mouse IgG HRP (1:200, Cwbio, Taizhou, China) at 37 °C for 30 min. Following the DAB staining, the sections were counterstained with hematoxylin and then were dehydrated and sealed in neutral resin. Positive signals of the tissues were observed under microscopy.

### 2.7. Quantitative Real-Time PCR (qRT-PCR)

Real-time quantitative polymerase chain reaction (qRT-PCR) analysis was performed to quantify the expression levels of ZO-1 and Occludin in using the SYBR^®^ Premix Ex Taq™ II (Tli RNaseH Plus) kit (Takara Biomedical Technology (Dalian) Co., Ltd., Dalian, China), with the normalization to β-actin. All experimental procedures strictly followed the standard protocol provided by the kit manufacturer. Relative gene expression levels were calculated using the 2^−ΔΔCt^ method (the primers are listed in Table 2).

## 3. Results

### 3.1. Identification of Morphology and Biochemistry of Strain

**Figure 1:**
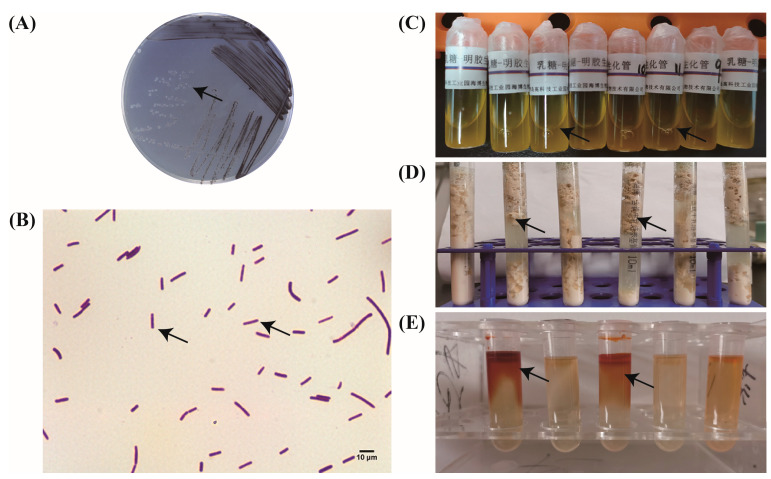
Isolation and biochemical identification of strains. (**A**) The round colonies on the Tryptose–Sulfite–Cycloserine (TSC) plate with black centers and smooth and gray edges (arrow). (**B**) Gram staining showing the Gram-positive strain with the short rod shapes in a single or ligation manner (arrow). (**C**) Biochemical detection of lactose and gelatin, with the red medium changed to a yellow liquid(arrow). (**D**) Iron milk medium showing characteristic “stormy fermentation” with gas production and coagulated casein forming sponge-like structures (arrow). (**E**) Identification through nitrate culture medium, with the culture medium turned red after adding a deoxidizer (arrow).

Strains isolated from the 6-month-old dead alpaca were cultured on a TSC agar plate, and their morphology was observed. The results showed that the colony was round and black-centered with smooth and gray edges (Figure 1A). Then, the strain was inoculated and cultured in FTG medium, showing a linear shape.

The solution of the bacteria cultured overnight was coated on the slide, fixed, and then stained with Gram staining. The results show that the bacterium was Gram-positive and short rod-shaped with blunt round ends. Usually, two strains are connected together, and occasionally several strains are connected in series. However, no spores were observed in the stain (Figure 1B).

The lactose–gelatin biochemical test on the isolated strain showed that the medium in the biochemical tube changed from red to yellow after inoculation of the strain, indicating that the strain could decompose lactose. At the same time, after the biochemical tube was left at 4 °C for 2 h, the gelatin became liquid, indicating that the strain can liquefy the gelatin (Figure 1C).

When the isolated bacteria were inoculated in the iron milk medium, a large number of bubbles initially floated from the bottom, indicating that the bacteria could produce gas, and then, casein coagulation in the iron milk medium formed sponge-like substances and gradually floated to the surface, indicating that the bacteria could cause a “boiling fermentation phenomenon” of the iron milk medium (Figure 1D).

After overnight cultivation of the isolated strain in nitrate medium, the color of the medium immediately turned red upon the addition of a nitrate-reducing reagent, while the negative control remained unchanged (Figure 1E).

### 3.2. Identification of Strain by PCR

**Figure 2:**
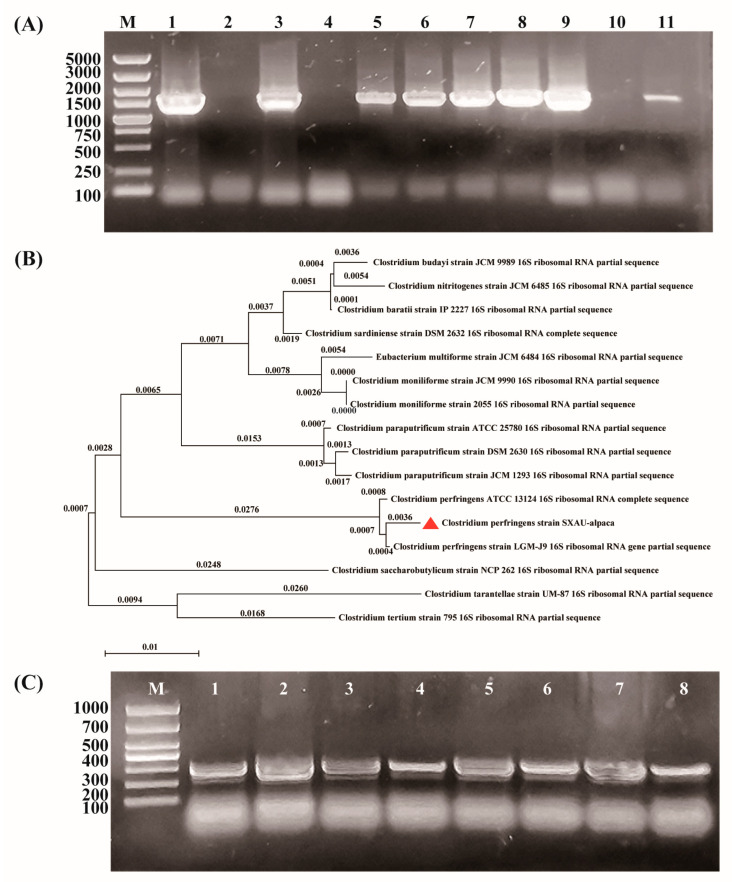
Identification of strain by PCR: (**A**) the agarose gel electrophoresis of *16S rRNA* amplification showing the target band with a size of 1500 bp; (**B**) phylogenetic analysis based on *16S rRNA* gene sequences indicating that the isolate strain (red triangle) close to strain LGM-J9; (**C**) the agarose gel electrophoresis of multiplex PCR of 6 toxins showing the only band of α toxin with a size of 300 bp.

Using the genomic DNA of the strain as a template, *16s RNA* was amplified and the agarose gel electrophoresis results showed a single band of 1500 bp (Figure 2A). After sequencing and Blast, it was identified as a *C. perfringens* strain. Multiple sequence alignment was carried out, and an evolutionary tree was constructed, which showed that the isolated strain shared high homology with the LGM-J9 strain (Figure 2B). Using the genomic DNA as a template, multiplex PCR with primers of the 6 kinds of toxin showed that only α toxin can be produced with a size of about 300 bp (Figure 2C). Therefore, the strain was preliminarily determined to be *C. perfringens* type A.

### 3.3. Pathogenicity of the Strain in Mice

**Figure 3:**
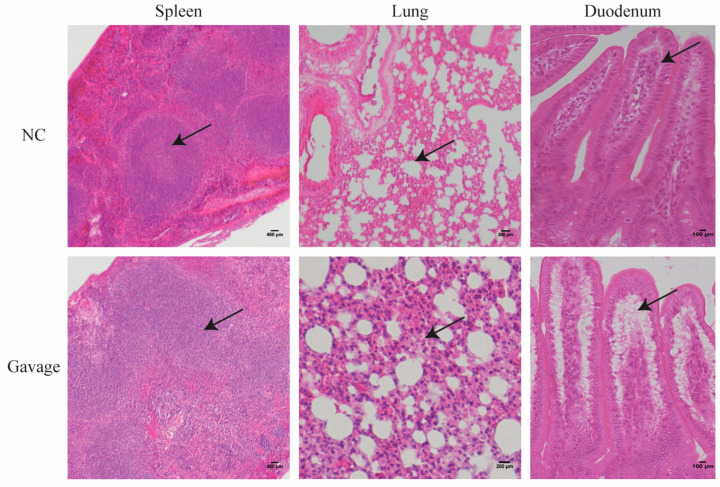
Pathological structure changes in tissues of mice infected by *C. perfringens* type A. The histological changes in the spleen, lungs, and duodenum were observed under a microscope after H & E staining. The NC (negative control) group were tissues from mice intragastrically gavaged with blank culture medium, and the Gavage group were pathological tissues from mice intragastrically gavaged with the culture supernatant of C. *perfringens* type A. The arrow represents the increased white pulp area in the spleen (40×), alveolar collapse and interstitial thickening (200×), and separated epithelial basement membrane and lamina propria of duodenum (200×) after H & E staining, respectively.

Twenty-four hours after the intragastric administration of 300 μL of the supernatant of *C. perfringens* type A, the mice showed disordered hair, dispiritedness, hyperphagia, and increased respiratory rates but no mice death. The mice were killed and dissected, and the pathological histology of the spleen, lungs, and duodenum were analyzed to determine the pathogenicity in mice. H & E staining showed that compared with NC mice, alveolar collapse and interstitial thickening of the lung, varying degrees of separation of the epithelial basement membrane and lamina propria of the duodenum, and significant increases and decreases in the area of the white and red pulp of the spleen occurred in the experimental mice (Figure 3).

### 3.4. Distribution of CD4^+^ and CD8^+^ T Cells in Spleen

**Figure 4:**
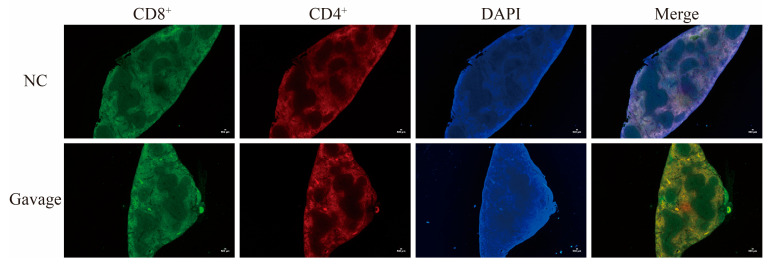
The distribution of CD4^+^/CD8^+^ T cells in the spleen of mice infected by *C. perfringens* type A. The NC (negative control) group were spleen from mice intragastrically gavaged with blank culture medium, and the Gavage group were pathological spleen from mice intragastrically gavaged with the culture supernatant of C. *perfringens* type A. CD4^+^ and CD8^+^ T cells were identified by red and green fluorescence, respectively. DAPI was used to identify cell nucleus (40×), purple and yellow signals in the merged images represent the colocalization of the indicated proteins.

The results of IFC with CD4^+^ and CD8^+^ T cells showed the presence of a positive signal in the tissue around the white pulp of the mouse spleen. Compared with the NC group, stronger staining of CD4^+^ T cells was observed in the red pulp of the spleen, and a weaker distribution of CD8^+^ T cells appeared in the red pulp of the spleen in mice with strain by intragastrical gavage (Figure 4).

### 3.5. Effect of C. perfringens Type A Strain on the Localization and mRNA Expression of *ZO-1* and *Occludin* in Duodenum Villus

**Figure 5:**
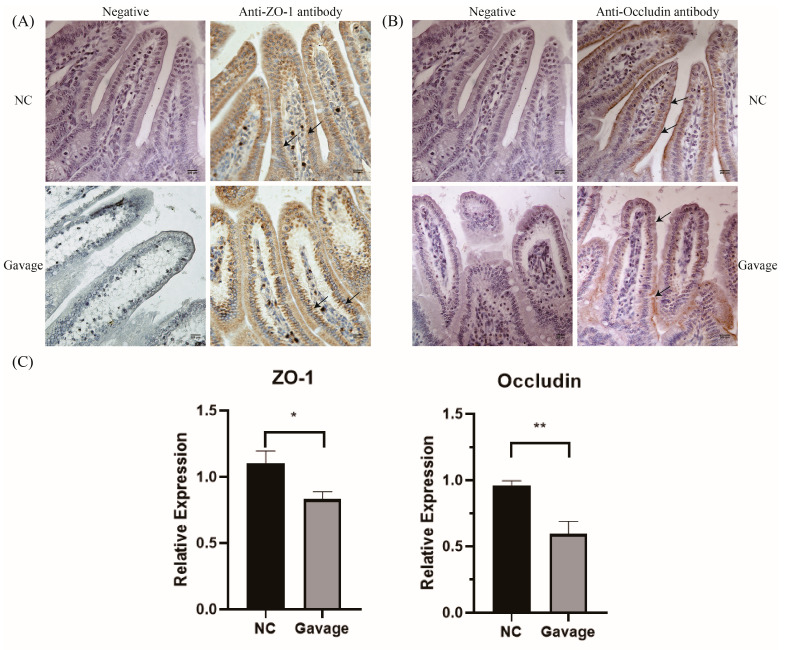
The effects of *C. perfringens* on the localization of ZO-1 and Occludin protein in the duodenum villus of mice (400×). (**A**) The localization of ZO-1 in the mucosa epithelium of the duodenum villus, with arrows representing the positive signal of ZO-1. (**B**) The localization of Occludin in the mucosa epithelium of the duodenum villus, with arrows indicating the positive signal of Occludin in cells. The NC group consists of the duodenum villus of mice that was gavaged with blank culture medium, and the gavage group consists of the duodenum villus of mice that was gavaged with culture supernatant. Brown staining indicates positive immunoreactivity, and nuclei were counterstained with hematoxylin (blue). (**C**) qRT-PCR result showing the mRNA expression levels of ZO-1 and Occludin with the normalization to *β-actin* in duodenum of mice decreased in the Gavage group, compared to those in the NC group with significant difference (*n* = 3, * *p* < 0.05, ** *p* < 0.01). The NC group represents the duodenum from mice intragastrically gavaged with blank culture medium, and the Gavage group represents the pathological duodenum from mice intragastrically gavaged with the culture supernatant of C. *perfringens* type A.

The results of IHC with ZO-1 showed that ZO-1 protein was localized in cytoplasma evenly in the mucosa epithelium of the duodenum villus of the mice in the control group. Additionally, compared with the control group, ZO-1 protein was mainly and weakly distributed in the free surface of the mucosa epithelium of the duodenum villus of mice infected by *C. perfringens* type A. Furthermore, the mucosa epithelium of the duodenum villus of the experimental mice were arranged in a relatively disordered manner (Figure 5A). The results of IHC with Occludin showed that the expression of Occludin in the duodenum of the mice with intragastric administration was relatively reduced and dispersed in the mucosa epithelium of the duodenum villus, and the expression of Occludin in the lateral border of the intestinal villi was less, being more concentrated near the intestinal gland (Figure 5B). The results of qRT-PCR showed that mRNA expression levels of ZO-1 and Occludin in duodenum of mice in Gavage group were decreased with significant difference (*p* < 0.05 or *p* < 0.01), compared to that in NC group (Figure 5C).

## 4. Discussion

*C. perfringens* is a Gram-positive bacterium and widely exists in soil, dust, fecal matter, feed, poultry litter, intestinal contents, and other environments. It is a common pathogen for humans and animals [9,10] and is associated with various important systemic and intestinal diseases, including gangrene, food poisoning, and necrotizing enteritis [11,12]. The pathogenicity of *C. perfringens* mainly comes from the four toxins [13]. Recently, death by *C. perfringens* has been reported in goats, turkeys, and other animals in India, Iran, Italy, and other countries [14,15,16]. In Xinjiang, China, there have been cases of pig infections [17].

*C. perfringens* was also isolated from the tissues of dead alpacas in Yangzhou Zoo in 2020 [18]. In this study, the strain was isolated from the intestinal content of a dead alpaca, showing the typical morphology and biochemistry characteristics of *C. perfringens*. However, during Gram staining, a small number of strains were found to be negative. The rupture of strains with long culture times has been reported to cause changes in the components of the bacterial cell wall, leading to false negatives. Similarly, we did not observe spore production from the Gram staining, and some researchers also found similar results for strains isolated from the human body [19].

In bacteria, the conserved region of *16S rRNA* can largely reflect the genetic relationship between bacterial species, while hypervariable regions reflect the differences between bacterial species. In this study, *16S rRNA* was amplified, the sequences were aligned with the NCBI database, and the results showed 99.99% homology with *C. perfringens*. Furthermore, the *16S rRNA* of the isolated strain and some known Clostridium species were used to establish an evolutionary tree, which showed that the isolated strain had high homology with LGM-9J. LGM-9J was isolated from piglets in a certain region of China, which suggested that *C. perfringens* might have been prevalent across the country and among multiple species, but this needs further research. Furthermore, the isolated strain was identified based on the PCR method for the toxins. Given the amplification of CPE and Net B with no bands. It corresponded with the Net B, which is produced by avian-derived type A strains and has not been detected in 32 non-avian-derived strains [8]. And the analysis of *C. perfringens* isolated from yak revealed that only type F produces CPE [20]. Therefore, CPA was specific for type A identification. But the isolating *C. perfringens* from a single dead alpaca limits the generalization of findings to regional prevalence.

To investigate the pathogenicity of *C. perfringens* type A, the supernatant of the cultured strain was gavaged into mice. The histological structure showed obvious separation of the intestinal epithelial basement membrane from the lamina propria, which might be the structural base for damage to the intestinal tract, resulting in “bloody intestines” in severe cases. Also, the white pulp area of the spleen in the mice was significantly expanded, which might be caused by the strong immune response of the mice through decreased CD4^+^ and increased CD8^+^ T cells.

Intestinal barrier function has become one of the key indicators to evaluate intestinal injury. Tight junctions are important in maintaining and regulating intestinal barrier function. The intestinal epithelial barrier consists of intestinal epithelial cells and their tight junctions (TJ) [21]. TJs, the most apical components of junctional complexes, are composed of multiple proteins, including the transmembrane proteins Occludin and claudins, and the peripheral membrane proteins zonula occludens-1 (ZO-1) and ZO-2 [22,23,24]. The proteins Occludin and ZO-1 have been shown to play important roles in the maintenance of TJ structure and epithelial barrier function [25]. Here, the expression of ZO-1 and Occludin on the duodenal mucosa of mice with strains isolated by gavage was decreased to varying degrees, indicating that the strain damaged the intestinal mucosal barrier. The damage to the intestinal mucosal barrier reduced the absorption of nutrients, resulting in growth retardation and poor fattening of the livestock and poultry. At the same time, α toxin and bacteria entered the blood, causing toxemia and bacteremia. These toxic and harmful substances reached the whole body through blood circulation, inducing pathological changes in various organs. Though the injecting bacterial culture supernatant into mice does not fully replicate the natural infection process of *C. perfringens*, it supplied the theoretical basis for the assessment of the pathogenicity of *C. perfiingens* type A. However, it was also fully recognized that the culture supernatant contains other secreted factors except toxin; thus, the potential synergistic effects of other virulence factors cannot be completely excluded, which needs further research.

## 5. Conclusions

The isolated strain of *C. perfringens* from the intestinal contents of an alpaca was type A. The pathogenicity of *C. perfringens* type A verified its lethal effects on the spleen, lungs, and duodenum through CD4^+^ and CD8^+^ T cells, and the expression of ZO-1 and Occludin. The results might be conducive to the development of precise drugs and vaccines resistant to *C. perfringens* type A.

## Figures and Tables

**Table 1:** Primers used for PCR.

Primers	Primer Sequence (5′-3′)	PCR Product (bp)
α-F	GCTAATGTTACTGCCGTTGA	325
α-R	CCTCTGATACATCGTGTAAG	
β-F	GCGAATATGCTGAATCATCTA	196
β-R	GCAGGAACATTAGTATATCTTC	
ε-F	GCGGTGATATCCATCTATTC	656
ε-R	CCACTTACTTGTCCTACTAAC	
ι-F	ACTACTCTCAGACAAGACAG	446
ι-R	CTTTCCTTCTATTACTATACG	
16S-F	AGAGTTTGATCCTGGCTCAG	1500
16S-R	TACGGCTACCTTGTTACGACTT	
CPE-F	TAACAATTTAAATCCAAT GG	233 [7]
CPE-R	ATTGAATAAGGGTAATTTCC	
Net B-F	TTTTCTTTTAGACATGTCCATAGGC	384 [8]
Net B-R	CCATCCCTTATTTCATCAGCATTTA	

**Table 2:** Primers used for *qRT-PCR*.

Primers	Primer Sequence (5′-3′)
ZO-1-F	GCCGCTAAGAGCACAGCAA
ZO-1-R	GCCCTCCTTTTAACACATCAGA
Occludin-F	TTGAAAGTCCACCTCCTTACAGA
Occludin-R	CCGGATAAAAAGAGTACGCTGG
β-actin-F	TTGCTGACAGGATGCAGAAG
β-actin-R	ACATCTGCTGGAAGGTGGAC

## Data Availability

Data are contained within the article.
